# Social Support and Disease Acceptance in Patients with Diabetic Foot Syndrome and Their Relationship with the Metabolic Control of the Disease

**DOI:** 10.3390/jcm14103412

**Published:** 2025-05-13

**Authors:** Ewa Kobos, Olga Serafin, Ewa Kostrzewa-Zabłocka, Anna Stefanowicz-Bielska

**Affiliations:** 1 Department of Development of Nursing, Social and Medical Sciences, Faculty of Health Sciences, Medical University of Warsaw, 61 Żwirki and Wigury Street, 02-091 Warsaw, Poland; 2 Faculty of Health Sciences, Medical University of Warsaw, 61 Żwirki and Wigury Street, 02-091 Warsaw, Poland; olga.serafin@interia.pl; 3 Diabetes Outpatient Clinic, Independent Public Provincial Specialist Hospital, 1 Ceramiczna Street, 22-100 Chełm, Poland; ewak@post.pl; 4 Division of Internal and Pediatric Nursing, Institute of Nursing and Midwifery, Faculty of Health Sciences with the Institute of Maritime and Tropical Medicine, Medical University of Gdansk, 80-211 Gdansk, Poland; ania-stefanowicz@gumed.edu.pl

**Keywords:** diabetes mellitus, diabetic foot, adaptation, social support, metabolic control

## Abstract

**Background**: Diabetic foot syndrome (DFS) constitutes a serious clinical challenge in the treatment of diabetes. The aim of this study was to assess social support and acceptance of the disease in patients with diabetic foot syndrome and their relationship with the metabolic control of diabetes. **Methods**: This was an observational, single-center study, conducted in 80 people hospitalized in the general and vascular surgery department. This study included adult patients with type 1 or type 2 diabetes, diagnosed with DFS. The mean age of the patients was 65.63 years, with the median age of 62 years. The youngest patient was 27, and the oldest was 94 years old. Men constituted 71.25% of the study group, women 28.75%. The following data were collected: the results of laboratory tests and measurements, the Acceptance of Illness Scale (AIS), and the Social Support Scale (S4-MAD) scores. **Results**: Abnormal values of non-high-density lipoprotein cholesterol (mean (M) = 120.76 mg/dL) and low-density lipoprotein cholesterol (M = 144.56) were shown in all the patients. Abnormal low-density lipoprotein values occurred in 98.75% of the patients (M = 148.21 mg/dL), and 83.75% of the participants had abnormal values of the systolic pressure (M = 145 mmHg) and total cholesterol. Glycated hemoglobin was abnormal in 61.25% of the subjects (M = 8.95%). The average score on the Acceptance of Illness Scale was 18.4 points in the study group. Out of the 100 possible points in the subscales of social support, the patients obtained an average of 46.5 points in the nutrition dimension, 40 for physical activity, 47.1 for glycemic self-control, 27.4 for foot care, and 68.9 for smoking. **Conclusions**: Patients with diabetic foot syndrome are characterized by poor acceptance of the disease and receive moderate social support. Patients receive the highest support in terms of cigarette smoking and glycemic self-control, with the lowest in foot care. The patient’s acceptance of the disease and the social support received are unrelated to the patient’s goals of disease control. Higher social support received by the DFS patients is associated with a greater acceptance of the disease.

## 1. Background

According to data from the International Diabetes Federation (IDF), the estimated number of people with diabetes aged 20–79 was 10.5% (536 million) in 2021. Projections for 2045 estimated an increase of this number to 12.2% [1]. Diabetic foot syndrome (DFS) is one of the most serious long-term complications of diabetes mellitus. It is characterized by the presence of neuropathy, ischemia, and an increased susceptibility to infections [2]. DFS is defined as an infection, ulceration, or destruction of the deep tissues of the foot, including bones, caused by damage to the peripheral nerves and/or blood vessels of the foot of varying severity [3,4]. A meta-analysis has shown that the overall prevalence of DFS was 9%, while the incidence was 4% [5]. It was estimated that over half of diabetic foot ulcers (DFU) became infected, and about one-fifth of individuals with DFU might require lower limb amputation [6]. The risk of death was found to be over two-fold in people with diabetes and DFU compared to those without ulcers [7]. Global statistics have indicated that the risk of developing foot ulcers in the lifetime of a person with diabetes ranged from 10% to 25%. A quarter of the patients were found to experience foot complications [2].

DFS prophylaxis is undoubtedly one of the most important aspects in the course of diabetes and includes the maintenance of metabolic balance by the patient and monitoring lipid parameters [3,8,9]. Metabolic balance criteria are also determined by self-blood glucose monitoring (SBGM), glycated hemoglobin (HbA1c), or time in range in patients using continuous glucose monitoring (CGM) [9]. The goals of balancing carbohydrate metabolism focus on adjusting HbA1c concentrations to the specific needs of different groups of patients. As regards the management of lipid metabolism, it is important to adjust the concentrations of LDL-C, non-HDL, HDL-C, and triglycerides to reduce cardiovascular risk, which is particularly high in people with diabetes [3]. Good metabolic control of diabetes requires the patients to be consciously involved in the treatment process and to regularly monitor their own health [3,10].

In addition to biomedical factors, psychosocial aspects, behavioral skills, and traits play an important role in the course of DFS [11,12]. The occurrence of DFS significantly affects the mobility of the patients, limiting their ability to move and perform everyday activities. This may contribute to social isolation, depression, and a decrease in the quality of life [13,14,15]. Ozyalcin and Sanlier found correlations between the acceptance of diabetes, depression, the quality of life, and emotional stress [16]. It was confirmed that, with the decreasing acceptance of the disease, the number of manifestations on the feet increased in people with type 2 diabetes treated in primary care facilities [17]. According to Akça Doğan et al., the degree of disease acceptance was related to the risk of developing the diabetic foot and the levels of fasting glucose and glycated hemoglobin in diabetic patients [18]. A significant relationship was demonstrated between the acceptance of diabetes and health-related behaviors undertaken by the patients [19].

Social support from the family, friends, or available support networks were indicated in the literature as the main factors facilitating the patients’ self-treatment for diabetes [12,20]. Social support may be understood to be the resources available to people in difficult or stressful circumstances. In medical and health sciences, it is seen to be the available support for the individual through social ties with other people, social groups, or the community. Social support is, quite broadly, any resource that flows between people. Social support may be understood to be any resource that flows through and from social relationships [21]. It was confirmed that the lack of active participation of the family in the self-control of a patient’s glycemia was observed in as many as 75.2% of diabetics, which directly translated into the patients’ mental and physical condition [22]. One study showed that only 1.8% of patients sought medical care in hospitals within 24 h of the onset of DFS-related symptoms. Patients with low social support and negative perceptions of the disease reported to medical facilities significantly later [23]. Research results showed that insufficient support might lead to poorer glycemic control, which increased the risk of complications [24,25]. People with diabetes who experienced strong social support were more likely to have better mental health, lower levels of stress, depression, and anxiety, as well as greater life satisfaction [26,27]. In addition to family support, social support is also important, which may include access to support groups, psychological counseling, as well as health education that helps the patient understand the disease and learn to cope with it [15,28,29]. The literature review has showed that the psychosocial aspects of the functioning of people with DFS and their relationship with the metabolic control of the disease were the subject of few studies for this group of patients. A qualitative study was conducted to examine the socio-cultural aspects of diabetic foot syndrome in a group of Italian patients hospitalized in a vascular surgery ward. Several key topics were identified that should be addressed when providing care to the patient, including the patient’s awareness of the diabetic foot, the life of the patient with the diabetic foot (the patient’s role in the family, body image, emotional state), the patient’s capacity of performing work, and the costs associated with the occurrence of the diabetic foot, barriers related to healthcare and ways of managing the diabetic foot at home, and implementing alternative medicine [30].

## 2. Aim

This study aims to assess the social support and acceptance of the disease in patients with diabetic foot syndrome, and their relationship with the metabolic control of diabetes.

## 3. Methods

### 3.1. Design and Setting of the Study

**Figure 1:**
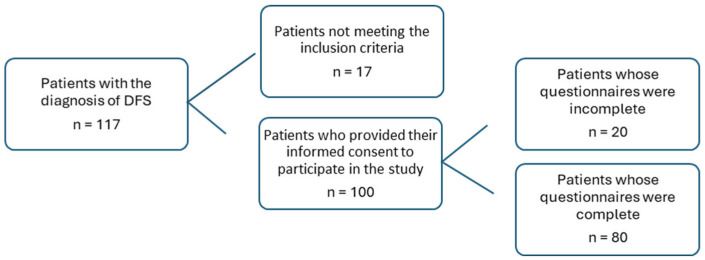
Study sample.

This was an observational, single-center cross-sectional study, conducted in the general and vascular surgery department on the day of hospital admission. The data were collected over a period of 6 months. At that time, 117 patients diagnosed with DFS were hospitalized in the department. In this group, 17 patients did not meet the criteria for inclusion in this study. Ultimately, 80 patients participated in this study (Figure 1).

### 3.2. Group Selection Criteria for the Study

The following patients were included in this study: adults, hospitalized patients with type 1 or type 2 diabetes, diagnosed with diabetic foot syndrome, a health condition allowing for participation in this study, and patients who gave their written consent to participate in this study. Patients who were unable to provide informed consent to participate in the study due to mental or cognitive impairment, who refused to participate, or who immediately received amputation were excluded from this study.

### 3.3. Research Tools

In order to collect the research material, we used the following tools, outlined in the below section.

#### 3.3.1. Results of Measurements and Laboratory Tests

This analysis took account of the results of the following measurements: body weight, height, body mass index (BMI), systolic and diastolic blood pressure values; laboratory test results from the patient’s medical records: glycated hemoglobin (HbA1c) concentration, and lipid concentration. The selection of clinical indices for analysis resulted from the recommendations of the Polish Diabetes Association (PDA) regarding the criteria for the metabolic control of diabetes [31].

#### 3.3.2. Acceptance of Illness Scale (AIS)

The Acceptance of Illness Scale was originally developed by Felton and Revenson [32]. The Polish adaptation was developed by Juczyński [33]. It includes eight elements describing the negative consequences of poor health. The scale is used to measure the degree of acceptance of the disease, which manifests itself in a lower intensity of negative reactions and emotions related to the present disease. On examination, the patient determines his current condition on a scale from 1 to 5, where 1 means that the patient strongly agrees with a statement, while 5 means that he strongly disagrees with a given statement. Strong agreement translates into poor adaptation to the disease; strong disagreement means acceptance of the disease. The total of all points is an overall measure of disease acceptance and ranges from 8 to 40 points. The study results were grouped into the following degrees of acceptance: <30 points—poor acceptance of the disease; 30–34 points—average acceptance of the disease; 35 and above—very good acceptance of the disease. The Cronbach’s alpha coefficient for this scale is satisfactory at 0.82 [32,33]. In this study, it was 0.927.

#### 3.3.3. Standardized Social Support Scale (S4-MAD)

The S4-MAD scale consists of 30 questions, with answers on a 5-point frequency scale: 1—never; 2—rarely; 3—occasionally; 4—often; 5—always. The scale includes five subscales: nutrition (10 questions), physical activity (5 questions), foot care (6 questions), smoking (3 questions) and glycemic control (9 questions). According to the key for the interpretation of the results, the patient may receive a maximum of 100 points in each subscale; the higher the score, the higher the social support received by the patient. The original scale is characterized by good internal consistency, with the Cronbach’s alpha coefficient being equal to 0.94 [34]. In a study by Ciemińska and Kobos, it equaled 0.93 [35]. In this study, the Cronbach’s alpha coefficient for the entire scale was 0.962; for the nutrition subscale, it was 0.845; for physical activity, it was 0.841; for glycemic self-control, it was 0.927; for foot care, it was 0.949; for smoking, it was 0.877.

### 3.4. Characteristics of the Study Group

In the study group, men accounted for 71.25%, with 63.8% of people being in a relationship, 40% having secondary vocational and general education, 40% working, and 27.5% receiving a disability benefit. A total of 81.25% of the patients inhabited urban areas. The average age of the participants was 65.63 ± 19.15 years (Me = 62). In the study group, people with type 2 diabetes accounted for 93.75%. Treatment with oral antidiabetic drugs was reported by 42.50% of them, and 41.25% had a lower limb amputated. The main reasons for hospitalization included pain in the lower limb (15%) and a non-healing wound in the lower limb (40%). The average duration of diabetes in the study group was 15.1 ± 9.06 years (Me = 14) (Table 1).

### 3.5. Statistical Analysis

The analysis was carried out with the use of Statistica 13.1. The Shapiro–Wilk test was used to examine the distribution of variables. The non-parametric Kruskal–Wallis test was used to characterize differences between more than two groups. The Spearman’s rank correlation test was also used, with the obtained values ranging from −1 to +1. Additionally, an exploratory multiple linear regression analysis (the “enter” method) was conducted to evaluate the impact of social support and clinical variables on metabolic control (HbA1c) and illness acceptance (AIS). A *p*-value of <0.05 was considered statistically significant. In the multiple regression analysis, listwise deletion was applied. Due to a high percentage of missing data, the smoking cessation variable was not included in the analysis.

A moderate correlation (r = 0.3) was detected at a significance level of α = 0.05, and the assumed test power of 80% (standard in socio-medical research) with the required sample size being about 87 people. Therefore, the size obtained in this study (80 individuals) is very close to the required value and allows for the detection of moderate effects with satisfactory statistical power.

## 4. Results

### 4.1. Metabolic Control Indices of Diabetes

The mean HbA1c concentration was 8.95 ± 2.48% in the study group. Triglycerides and non-HDL cholesterol had high mean values of 250.81 ± 115.31 mg/dL and 120.76 ± 32.96 mg/dL, respectively. A summary of metabolic control indices of diabetes is presented in Table 2.

**Figure 2:**
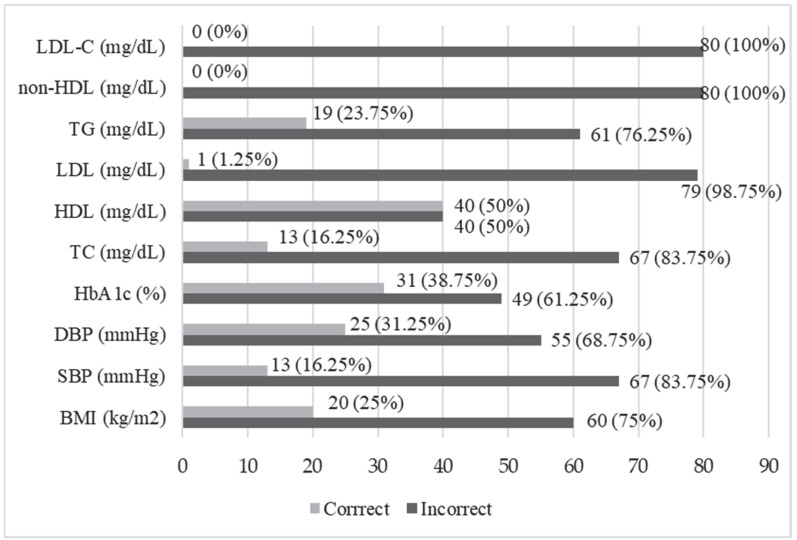
Distribution of the respondents based on the normality of the values of metabolic control indices of diabetes. BMI, body mass index; SBP, systolic blood pressure; DBP, diastolic blood pressure; HbA1c, glycated hemoglobin; TC, total cholesterol; HDL, high-density lipoprotein cholesterol; LDL, low-density lipoprotein cholesterol; TG, triglycerides; non-HDL, non-high-density lipoprotein cholesterol; LDL-C, low-density lipoprotein cholesterol.

The data showed that all the patients had abnormal values of non-HDL and LDL-C. Abnormal values of LDL occurred in 98.75% of the patients, and 83.75% of the participants had abnormal values of the systolic pressure and total cholesterol. The HbA1c index was abnormal in 61.25% of the subjects (Figure 2).

### 4.2. Acceptance of Illness

The average score on the Acceptance of Illness Scale was 18.4 ± 7.1 points (Me = 17) in the study group. The scores ranged from 8 to 37 points. Very good acceptance of the disease was demonstrated in 2.5% of the patients, while people with poor acceptance of the disease constituted 92.5% of the total study group (Table 3). The answers of the respondents regarding the AIS are reported in Supplementary Materials (Table S1).

The analyses showed that most correlations between the results of the Acceptance of Illness Scale and the metabolic control indices of diabetes control were weak and did not reach the level of statistical significance (*p* > 0.05). A significant negative correlation was confirmed between triglyceride concentrations and disease acceptance (ρ = −0.236; *p* = 0.035) (Table 4).

### 4.3. Social Support

In the study group, the highest mean value was obtained by the patients in the social support subscale of smoking 68.9 ± 24.8 (Me = 75.0), while the lowest scores were noted in the subscale of foot care 27.4 ± 22.7 (Me = 25) (Table 5). The answers of the respondents regarding the S4-MAS scale are reported in Supplementary Materials (Table S2).

The analysis of the correlation between the overall score obtained by the patients on the Social Support Scale and most values of metabolic control indices of diabetes showed a value close to zero and did not reach the statistical significance of *p* > 0.05 (Table 6).

The analysis of the correlation between the acceptance of the disease and the social support obtained showed positive relationships of varying strength. The moderate strength of the relationship between disease acceptance and nutritional support (rho = 0.346; *p* = 0.002) and between disease acceptance and foot care subscale scores (rho = 0.318; *p* = 0.004) was confirmed. The strongest correlation (rho = 0.538; *p* < 0.001) was demonstrated between disease acceptance and social support received by the patients in general (Table 7).

### 4.4. Multiple Linear Regression Analysis

The predictors included the S4-MAD subscales (nutrition, physical activity, foot care, glycemic control), the overall social support score, and the Acceptance of Illness Scale (AIS) score (for the HbA1c regression only). The overall score was included despite its conceptual redundancy with the subscales to maintain consistency with the original correlational analyses (see: Table 6 and Table 7).

The regression analysis was performed solely for HbA1c as a dependent variable because metabolic control was the primary focus of this study, consistent with its original design. Additionally, age, type of diabetes, duration of diabetes, and the occurrence of amputations were included as covariates. All variables were entered simultaneously into the models (the “enter” method). Assumptions for multiple regression were verified, including the normality of residuals and the absence of multicollinearity (based on tolerance and VIF values).

The results of the regression analysis indicated that only the type of diabetes was a significant predictor of HbA1c levels (*p* = 0.012), whereas all other variables were not statistically significant (*p* > 0.05). The model explained approximately 26% of the variance in HbA1c levels (R^2^ = 0.262). These findings are consistent with the previous bivariate correlation analysis, which showed that the correlations between the overall social support score and most metabolic control indices of diabetes were close to zero and did not reach statistical significance (Table 8).

The results of the regression analysis indicated that only the presence of amputation was a significant predictor of the level of disease acceptance (*p* = 0.001). Other variables, including subscales of social support and clinical variables, did not reach statistical significance (*p* > 0.05). The model explained approximately 37.5% of the variance in the AIS scores (R^2^ = 0.375). These findings partly differ from the earlier bivariate correlation analysis, which showed positive relationships of varying strength between the level of disease acceptance and the social support subscales. A moderate correlation was observed between disease acceptance and nutritional support (rho = 0.346; *p* = 0.002) and between disease acceptance and foot care support (rho = 0.318; *p* = 0.004), while the strongest correlation was found between disease acceptance and the overall level of social support received (rho = 0.538; *p* < 0.001) (Table 9).

These findings suggest that clinical factors may have a stronger impact on metabolic control and illness acceptance than social support in the studied group of patients.

## 5. Discussion

The assessment of the degree of the metabolic control of the disease based on the indices included in the 2006 PDA guidelines was performed in a group of 1045 patients with type 2 diabetes. It showed that the concentration of triglycerides was the most frequently met compensation criterion (50.4% of patients), while the HbA1c value was met the least frequently (12.1% of patients). The authors also analyzed the compliance of the results with the 2011 PDA guidelines, where 66.9% of the patients met the criterion for the diastolic blood pressure, and 18.7% achieved a satisfactory concentration of the LDL cholesterol fraction [36]. The study showed that TG values were normal only in 23.75% of the patients with DFS, while no patients achieved normal non-HDL or LDL-C values. Abnormal LDL, DBP, and TC values occurred in about 90% of the subjects. The recommended HbA1c concentration was achieved by 38.75% of the patients. The results of this study indicated that there was significant room for improvement in the context of comprehensive metabolic control, and the results of systolic blood pressure and lipid level control showed much higher values than in patients with type 2 diabetes, taking account of the results published by Łagowska-Batyra et al. [36]. Intensive glycemic control (HbA1c 6.0–7.5%) was associated with a significant reduction in the risk of amputation [37]. Achieving such a goal requires continuous monitoring and the adaptation of treatment strategies in order to better adapt to the needs of the individual patients. This may significantly contribute to improving their quality of life and reducing the risk of developing and deteriorating the complications of diabetes, including DFS. In a 2018 study conducted in type 2 diabetics in a diabetes clinic, elevated levels of TC were confirmed in 60.7% of the patients, LDL fraction in 79.2%, DBP in 63.9%, and elevated HbA1c values in 44.8% [10]. In a study conducted in China, significant differences in the ratios between total cholesterol/high-density lipoprotein cholesterol and low-density lipoprotein cholesterol/high-density lipoprotein cholesterol were observed in patients with type 2 diabetes compared to patients with type 2 diabetes and DFS. The study also showed that the patients with type 2 diabetes and DFS were characterized by poorer glycemic control than the patients with type 2 diabetes [38]. A study by Ardelean et al. [39] showed that the end-stage disease might be indicated by lower HDL-C levels and better lipid profile in the patients with type 2 diabetes and infected diabetic foot ulcers compared to those patients with type 2 diabetes. Notably, the study included patients hospitalized in the vascular surgery department, and a high percentage of them had already undergone amputations in the lower limb or had been hospitalized due to a poorly healing wound in the lower limb.

Some authors have indicated a relationship between psychosocial factors and diabetes control [40]. Therefore, the aim of this study was to assess social support and acceptance of the disease in the patients with DFS and their relationship with metabolic control of the disease. This study showed poor acceptance of diabetes in patients with DFS. The average score obtained in this study (18.40) was lower than in the group of patients hospitalized with type 2 diabetes, excluding those with DFS, or patients with type 2 diabetes in primary care [16,17,41,42]. The average AIS score was 27.65 for hospitalized patients after lower limb amputation due to DFS, confirming low disease acceptance [43]. In this study, 41.24% of the patients had their lower limb amputated. The AIS was used to assess disease acceptance in the patients with DFS after lower limb amputation in two studies in Poland. The first one confirmed that the level of acceptance of the disease was lower in individuals experiencing greater pain, and the second one confirmed that it was higher in those with a better quality of life [43,44].

The study did not confirm a significant association between disease acceptance and metabolic control scores achieved by the patients, except for higher TG values in the patients with lower acceptance levels. Also, a study by Rusin-Pawełek et al. [41] did not confirm the relationship between diabetes control indices, such as BMI, HbA1c concentration and the degree of disease acceptance. Akça Doğan et al. reported that higher acceptance of the disease in patients with type 2 diabetes was correlated with lower fasting glucose and HbA1c concentrations, and a lower risk of DFS [18].

The overall level of social support received in the studied group of patients with DFS was moderate and varied depending on the area of self-care for diabetes. The patients received the highest support in terms of cigarette smoking and glycemic self-control, while the lowest in the case of foot care. Although the S4-MAD questionnaire is a tool designed to measure social support in the self-care of middle-aged (30–60 years) people with type 2 diabetes, in this study it was first used to assess support in the group of people with type 1 and 2 diabetes, with diabetic foot syndrome and over 60 years of age. Therefore, the present results may only be applied to those obtained in patients with type 2 diabetes. Similar findings were published by Ciemińska and Kobos [35] regarding a group of outpatients with type 2 diabetes. The data showed that diabetic foot self-care behaviors were at a moderate level [29], and treatment failure might be associated with patient non-compliance. Effective treatment for diabetes depends on the ability and willingness of the patients to adhere to treatment and practice self-care behaviors, and this may be related to the level of acceptance of the disease [17]. Taking account of the fact that this study included patients with already diagnosed DFS, and that many of them had undergone amputations of a lower limb, the social support received from relatives or medical staff may have been insufficient. This may confirm that those persons in the patients’ environment should be more involved than before by medical staff in diabetes education. Moreover, the awareness of the importance of supporting a close person in regular foot inspection and care should be increased. The availability of a social support network should be analyzed with the patients in clinical practice and developed with the use of various sources of support. In a study by Werfalli et al. [44,45], 75% of the people with type 2 diabetes over 60 years of age believed that their family supported them in complying with all aspects of self-care management. Low social support was recorded in 23.2% of the participants. Greater social support received by the patients was associated with greater self-care, as shown by Ampofo et al. [46]. The mean S4-MAD score obtained in the study by Ampofo et al. was 54.27 ± 16.37. Notably, the research was carried out in culturally different conditions, where the direct involvement of family members in caring for the sick or elderly might be greater than in European countries.

The analysis of the results of this study showed that the level of social support received did not significantly correlate with the metabolic control indices of diabetes in the patients with DFS, which may indicate the lack of a direct relationship between social support and those indices. This study did not confirm the relationship between support regarding the smoking subscale and metabolic control of diabetes in people with DFS. However, according to the literature, cigarette smoking and glycemic control are important factors in the formation and progression of lesions in the course of DFS [2,5]. Mohebi et al. [47] demonstrated an association between better social support and self-care behaviors in patients with type 2 diabetes. Patients with higher HbA1c levels experienced lower levels of social support. However, the finding was statistically insignificant.

The lack of psychosocial support was commonly indicated by diabetic patients [48]. Scientific data confirmed that support was a social factor that affected glycemic control to varying degrees in people with diabetes [20,39,49,50,51,52,53]. Lu et al. reported a tendency to higher levels of blood glucose and HbA1c, TG, TC, low- and high-density lipoproteins in middle-aged men with diabetes and experiencing greater social isolation [54]. According to Ozturk et al., as the social support levels reported by the patients increased, self-care behaviors also increased as regards diet, physical activity, glucose level monitoring, foot care, and the total score of diabetes self-care activities scale [55]. As regards the primary care of the patients with uncontrolled type 2 diabetes, higher HbA1c concentrations were associated with reduced social bonds [56]. However, there are conflicting study results indicating no association between social support and HbA1c levels and high social support contributing to poor glycemic control [40,57]. Some authors have claimed that higher social support was associated with higher TC, LDL, and TG levels [55]. Another study confirmed that social support was a predictor of physical activity. The chances of minimal physical activity in the patients increased as social support grew [58].

Higher social support received by the DFS patients was associated with greater acceptance of the disease. One study, including people with type 2 diabetes (without the diagnosis of DFS), showed that family support had a positive effect on disease acceptance, metabolic control and adherence [42].

Costa et al. emphasized the need to integrate scientific data from social sciences into all aspects of the care of patients with DFS (biomedical, behavioral, social, and cultural aspects). The integrated model proposed by the authors is based on the relationship between biomedical aspects and social sciences, in order to improve clinical outcomes and satisfaction in the patients with DFS. This requires the involvement of not only healthcare professionals but psychologists, sociologists, or anthropologists in the care of patients [30].

## 6. Study Limitations

This study has several limitations. The cross-sectional design of this study prevents the establishment of a cause-and-effect relationship, limiting the strength of the conclusions. The participants of this study had DFS and were hospitalized, which may not be representative of all patients diagnosed with DFS. The sample size was also restricted due to specific enrollment criteria, which may affect the generalizability of the findings. This was a single-center study, carried out in a small sample of patients, which limits the generalizability of the results. A formal calculation of the sample size had not been carried out prior to this study, which is a methodological limitation. However, due to the nature of this study (exploratory, cross-sectional) and the use of all the available patients meeting the criteria within a certain timeframe, the obtained sample size provided sufficient power to detect moderate-sized effects. The sample size was adequate to the purpose and nature of this study.

## 7. Conclusions

In this study, the incidence of poor glycemic control observed in the patients with DFS was significantly high and the disease acceptance was low. The patient’s acceptance of the disease and the social support received are unrelated to the patient’s goals of disease control. Medical staff should periodically assess the acceptance of the disease in this group of patients. The link between social support and disease acceptance in people with DFS highlights the importance of strengthening social networks to increase disease acceptance. When providing care, healthcare professionals should assess the availability and quality of the social support, encouraging the patients to seek and maintain supportive relationships with their family and friends. The significant role of family members and caregivers in providing support should be taken into account in planning the care of a patient with DFS. The complexity of ailments associated with DFS does not only include the values of clinical indices achieved by the patients but includes the values concerned with the psychological and social sphere.

## Figures and Tables

**Table 1:** Characteristics of the study group (n = 80).

Variable		%
Sex	Woman	28.75
	Man	71.25
Marital status	In a relationship	63.8
	Single	36.2
Education	Primary or lower secondary school	6.25
	Basic vocational education	31.25
	Secondary or post-secondary school	40
	Higher	22.5
Occupational status	Non-working	10
	Retirement or disability pension	50
	Workers	40
Place of residence	Town	81.25
	Village	18.75
Occurrence of amputations	Yes	41.25
	No	58.75
Type of amputations	Amputation of one or two toes	13.75
	Forefoot amputation	8.75
	Foot amputation	6.25
	Lower leg amputation	6.5
	Lower limb amputation	5.0
Type of diabetes	Type 1	6.25
	Type 2	93.75
Type of treatment	Antihyperglycemic medications	42.5
	Insulin	31.25
	Antihyperglycemic medications and insulin	26.25
Presence of chronic complications	Neuropathy	42.5
	Nephropathy	18.75
	Retinopathy	31.25
	Myocardial infarction	31.25
	Stroke	32.5

**Table 2:** Values of individual metabolic control indices of diabetes (n = 80).

Indices	M	Me	SD	Min	Max	Skewness	Kurtosis	Shapiro–Wilk test	
								W	*p*
BMI (kg/m^2^)	27.82	27.91	4.98	15.18	41.6	0.3193	0.8020	0.982	0.329
SBP (mmHg)	145.00	146.00	16.49	96	177	−0.4596	0.6299	0.968	0.045
DBP (mmHg)	85.26	88.00	12.78	42	112	−0.9562	1.3406	0.939	<0.001
HbA1c (%)	8.95	8.30	2.48	4.60	18.2	0.9404	1.6746	0.946	0.002
HDL (mg/dL)	48.95	42.00	23.69	27	199	3.9510	21.2362	0.629	<0.001
LDL (mg/dL)	148.21	148.00	32.75	42	201	−0.5315	0.0161	0.962	0.018
TG (mg/dL)	250.81	215.00	115.31	112	582	0.9735	0.4761	0.905	<0.001
non-HDL (mg/dL)	120.76	112.50	32.96	71	313	2.5866	13.3602	0.803	<0.001
LDL-C (mg/dL)	144.56	142.00	22.26	111	248	1.3733	4.4294	0.909	<0.001

M, mean; Me, median; SD, standard deviation; Min, minimum; Max, maximum; W, value of the Shapiro–Wilk test; *p*, statistical significance level; BMI, body mass index; SBP, systolic blood pressure; DBP, diastolic blood pressure; HbA1c, glycated hemoglobin; HDL, high-density lipoprotein cholesterol; LDL, low-density lipoprotein; TG, triglycerides; non-HDL, non-high-density lipoprotein cholesterol; LDL-C, low-density lipoprotein cholesterol.

**Table 3:** Acceptance of illness in the study group.

Acceptance of Illness	N	M	Me	SD	Min	Max	Skewness	Kurtosis	Shapiro–Wilk Test		Kruskal–Wallis Test		
									W	*p*	χ^2^	df	*p*
High acceptance	2	36.5	36.5	0.707	36	37	-	-	-	-	16.5	2	<0.001
Medium acceptance	4	32.5	32.5	1.291	31	34	0.000	−1.200	0.993	0.972			
Low acceptance	74	17.2	17.0	5.758	8	29	0.222	−0.740	0.966	0.043			
Total score	80	18.4	17.0	7.10	8	37	0.613	−0.111	0.957	0.008	-	-	-

M, mean; Me, median; SD, standard deviation; Min, minimum; Max, maximum; W, value of the Shapiro–Wilk test; df, degrees of freedom; *p*, statistical significance level.

**Table 4:** Correlations between the values of the metabolic control indices of diabetes and the Acceptance of Illness score.

Variable	Spearman’s ρ	Acceptance of Illness
BMI (kg/m^2^)	ρ	−0.130
	df	78
	*p*	0.249
SBP (mmHg)	ρ	−0.073
	df	78
	*p*	0.518
DBP (mmHg)	ρ	0.083
	df	78
	*p*	0.464
HbA1c (%)	ρ	0.009
	df	78
	*p*	0.938
TC (mg/dL)	ρ	−0.167
	df	78
	*p*	0.139
HDL (mg/dL)	ρ	−0.006
	df	78
	*p*	0.956
LDL (mg/dL)	ρ	−0.151
	df	78
	*p*	0.180
TG (mg/dL)	ρ	−0.236
	df	78
	*p*	0.035
non-HDL (mg/dL)	ρ	0.207
	df	78
	*p*	0.065
LDL-C (mg/dL)	ρ	−0.188
	df	78
	*p*	0.095

ρ, value of the Spearman’s test; *p*, statistical significance level; df, degrees of freedom; BMI, body mass index; SBP, systolic blood pressure; DBP, diastolic blood pressure; HbA1c, glycated hemoglobin; TC, total cholesterol; HDL, high-density lipoprotein cholesterol; LDL, low-density lipoprotein; TG, triglycerides; non-HDL, non-high-density lipoprotein cholesterol; LDL-C, low-density lipoprotein cholesterol.

**Table 5:** The results of the Social Support Scale broken down into individual subscales (n = 80).

S4-MAD Subscales	M	Me	SD	Min	Max	Skewness	Kurtosis	Shapiro–Wilk Test	
								W	*p*
Nutrition	46.5	44.4	18.6	2.78	100	0.218	0.749	0.979	0.223
Physical activity	40.1	40.0	23.8	0.00	100	0.483	−0.254	0.964	0.024
Self-monitoring of blood glucose	47.1	39.3	23.1	0.00	100	0.268	−0.409	0.967	0.038
Foot care	27.4	25.0	22.7	0.00	100	1.094	1.001	0.905	<0.001
Smoking (n = 39)	68.9	75.0	24.8	0.00	100	−1.531	2.079	0.799	<0.001

M, mean; Me, median; SD, standard deviation; Min, minimum; Max, maximum; W, value of the Shapiro–Wilk test; *p*, statistical significance level.

**Table 6:** Correlations between the values of metabolic control indices of diabetes and the social support obtained.

S4-MAD Subscales/Indices of Metabolic Control		BMI (kg/m^2^)	DBP (mmHg)	SBP (mmHg)	HbA1c (%)	TC (mg/dL)	HDL (mg/dL)	LDL (mg/dL)	Non-HDL (mg/dL)	TG (mg/dL)	LDL-C (mg/dL)
Nutrition	Spearman’s Rho	−0.086	0.000	−0.119	−0.127	−0.055	0.090	−0.065	0.107	−0.127	0.098
	df	78	78	78	78	78	78	78	78	78	78
	*p*	0.448	0.999	0.295	0.263	0.629	0.428	0.564	0.346	0.262	0.389
Physical activity	Spearman’s Rho	−0.001	0.132	0.001	0.046	0.040	0.105	−0.021	0.058	−0.036	0.099
	df	78	78	78	78	78	78	78	78	78	78
	*p*	0.990	0.244	0.995	0.683	0.726	0.354	0.856	0.608	0.749	0.384
Self-monitoring of blood glucose	Spearman’s Rho	−0.033	0.064	−0.021	−0.018	−0.042	0.080	−0.078	0.139	−0.087	0.021
	df	78	78	78	78	78	78	78	78	78	78
	*p*	0.771	0.571	0.852	0.877	0.709	0.478	0.494	0.220	0.441	0.851
Foot care	Spearman’s Rho	0.131	0.112	0.012	0.066	0.115	−0.080	0.072	−0.034	0.034	0.076
	df	78	78	78	78	78	78	78	78	78	78
	*p*	0.246	0.322	0.917	0.559	0.310	0.481	0.528	0.765	0.765	0.502
Smoking (n = 39)	Spearman’s Rho	0.054	0.012	−0.044	−0.060	−0.070	0.165	−0.086	0.151	−0.184	−0.094
	df	37	37	37	37	37	37	37	37	37	37
	*p*	0.742	0.941	0.789	0.718	0.670	0.315	0.605	0.358	0.263	0.571
Total	Spearman’s Rho	−0.006	0.081	0.002	0.014	0.030	0.010	−0.040	0.099	−0.083	0.060
	df	78	78	78	78	78	78	78	78	78	78
	*p*	0.958	0.475	0.984	0.904	0.789	0.930	0.726	0.383	0.464	0.596

Rho, Spearman’s correlation coefficient; *p*, statistical significance level; df, degrees of freedom; BMI, body mass index; SBP, systolic blood pressure; DBP, diastolic blood pressure; HbA1c, glycated hemoglobin; TC, total cholesterol; HDL, high-density lipoprotein cholesterol; LDL, low-density lipoprotein; TG, triglycerides; non-HDL, non-high-density lipoprotein cholesterol; LDL-C, low-density lipoprotein cholesterol.

**Table 7:** Correlations between the results of disease acceptance and social support.

Social Support (S4-MAD)		Acceptance of Illness (AIS)
Nutrition	Spearman’s Rho	0.346
	df	78
	*p*	0.002
Physical activity	Spearman’s Rho	0.243
	df	78
	*p*	0.030
Self-monitoring of blood glucose	Spearman’s Rho	0.302
	df	78
	*p*	0.006
Foot care	Spearman’s Rho	0.318
	df	78
	*p*	0.004
Smoking (n = 39)	Spearman’s Rho	0.019
	df	37
	*p*	0.910
Total social support	Spearman’s Rho	0.538
	df	78
	*p*	<0.001

Rho, Spearman’s correlation coefficient; *p*, statistical significance level; df, degrees of freedom.

**Table 8:** Results of multiple linear regression analysis for HbA1c levels.

	F-Statistics = 2.45 (*p* = 0.0146), R^2^ = 0.262, N = 80				
Variable	B	SE	Beta (Stand.)	t	*p*
Constant	43.20	15.24	—	2.83	0.006
Nutrition	0.048	0.381	0.050	0.13	0.900
Physical activity	0.029	0.249	0.035	0.12	0.908
Foot care	0.239	0.260	0.278	0.92	0.363
Self-monitoring of blood glucose	−0.066	0.313	−0.078	−0.21	0.834
Social support	0.032	1.006	0.032	0.03	0.975
Disease acceptance	−0.351	0.359	−0.128	−0.98	0.333
Age	−0.117	0.120	−0.114	−0.97	0.335
Type of diabetes	−26.29	10.14	−0.329	−2.59	0.012
Duration of diabetes	−0.066	0.241	−0.030	−0.27	0.786
Amputation	−1.63	4.71	−0.042	−0.35	0.729

B, unstandardized regression coefficient; SE, standard error; Beta, standardized regression coefficient; t, t-statistic value; *p*, level of statistical significance.

**Table 9:** Results of multiple linear regression analysis for the Acceptance of Illness Scale (AIS).

	F-Statistics = 4.67 (*p* = 0.000074), R^2^ = 0.375, N = 80				
Variable	B	SE	Beta (Stand.)	t	*p*
Constant	20.87	4.42	—	4.73	<0.001
Nutrition	0.036	0.127	0.102	0.28	0.779
Physical activity	−0.034	0.083	−0.115	−0.42	0.679
Foot care	0.071	0.086	0.225	0.82	0.416
Self-monitoring of blood glucose	0.011	0.104	0.036	0.11	0.916
Social support	0.061	0.335	0.017	0.18	0.856
Age	−0.036	0.040	−0.097	−0.91	0.366
Type of diabetes	−2.50	3.36	−0.086	−0.75	0.458
Duration of diabetes	−0.066	0.080	−0.085	−0.83	0.409
Amputation	−4.97	1.45	−0.347	−3.43	0.001

B, unstandardized regression coefficient; SE, standard error; Beta, standardized regression coefficient; t, t-statistic value; *p*, level of statistical significance.

## Data Availability

The datasets generated and/or analyzed during the current study are not publicly available due to confidentiality, but the data are accessible from the corresponding author upon reasonable request.

## Supplementary Materials

The following supporting information can be downloaded at: https://www.mdpi.com/article/10.3390/jcm14103412/s1, Table S1. Distribution of Responses to Individual Items of the Acceptance of Illness Scale (AIS). Table S2. Distribution of the Number (N) and Percentage (%) of Responses to Individual Items of the S4-MAD.

## List of Abbreviations

AIS	Acceptance of Illness Scale
BMI	body mass index
CGM	continuous glucose monitoring
DBP	diastolic blood pressure
DF	diabetic foot
DFU	diabetic foot ulcer
HbA1c	glycated hemoglobin
HDL	high-density lipoprotein cholesterol
LDL	low-density lipoprotein
LDL-C	low-density lipoprotein cholesterol
non-HDL	non-high-density lipoprotein cholesterol
PTD	Polish Diabetes Association
S4-MAD	Standardized Social Support Scale
SBGM	self-blood finger-stick glucose monitoring
SBP	systolic blood pressure
TC	total cholesterol
TG	triglycerides
