# Supplementary material for: Social Support and Disease Acceptance in Patients with Diabetic Foot Syndrome and Their Relationship with the Metabolic Control of the Disease
Source: J Clin Med. 2025 May 13;14(10):3412. doi: 10.3390/jcm14103412 (PMC12112424; doi:10.3390/jcm14103412)
Supplement: Supplementary file 1 [file jcm-14-03412-s001.zip › jcm-3569410-supplementary.pdf]

Table S1. Distribution of Responses to Individual Items of the Acceptance of Illness Scale (AIS).

Statement	Answers				
	1 – strongly agree n (%)	2 – agree n (%)	3 – I don't know n (%)	4 – disagree n (%)	5 – strongly disagree n (%)
I am having trouble adapting to the restrictions imposed by the disease	37 (46.25)	21 (26.25)	14 (17.50)	8 (10.00)	0 (0.00)
Because of my health condition, I'm not able to do what I like the most	27 (33.75)	21 (26.25)	20 (25.00)	11 (13.75)	1 (1.25)
The disease sometimes makes me feel unnecessary	19 (23.75)	30 (37.50)	15 (18.75)	14 (17.50)	2 (2.50)
Health problems make me more dependent on others than I want	28 (35.00)	29 (36.25)	12 (15.00)	9 (11.25)	2 (2.50)
The disease makes me a burden to my family and friends	17 (21.25)	26 (32.50)	22 (27.50)	14 (17.50)	1 (1.25)
My health makes me feel like I'm not a complete person	17 (21.25)	27 (33.75)	16 (20.00)	17 (21.25)	3 (3.75)
I will never be self-sufficient enough to the level I would like to be	31 (38.75)	21 (26.25)	14 (17.50)	13 (16.25)	1 (1.25)
I think the people around me are often embarrassed about my illness	11 (13.75)	22 (27.50)	29 (36.25)	15 (18.75)	3 (3.75)

n = number of participants; % = percentage of total responses.

Table S2. Distribution of the Number (N) and Percentage (%) of Responses to Individual Items of the S4-MAD.

Question	Answers				
I HAVE ….	1 – never n (%)	2 – rarely n (%)	3 – occasionally n (%)	4 – often n (%)	5 – always n (%)
1. Somebody who encourages me to stick to the diet recommended by my physician or nutritionist.	3(3.75)	24(30)	17(21.25)	32(40)	4(5)
2. Somebody who shows how happy she/he is when I stick to the diet by my physician or nutritionist.	3(3.75)	22(27.5)	16(20)	35(43.75)	4(5)
3. Somebody who buys the necessary ingredients to cook foods appropriate for diabetics.	3(3.75)	34(42.5)	17(21.25)	23(28.75)	3(3.75)
4. Somebody who helps me to schedule meals and snacks.	7(8.75)	31(38.75)	20(25)	20(25)	2(2.5)
5. Somebody who cooks foods appropriate for a diabetic patient for me.	4(5)	19(23.75)	27(33.75)	26(32.5)	4(5)
6. Somebody who warns me when I eat more or less than of my eating plan.	5(6.25)	34(42.5)	14(17.5)	23(28.75)	4(5)
7. Somebody who eats the foods that I can eat so that I do not have any temptation and can go on with my diet.	16(20)	27(33.75)	17(21.25)	17(21.25)	3(3.75)
8. Somebody who – before any meal or snack – tells me if the ingredients of that food are appropriate for me or not.	9(11.25)	39(48.75)	9(11.25)	19(23.75)	4(5)
9. Somebody who reminds me repeatedly about the necessity of continuing my diet.	10(12.5)	21(26.25)	13(16.25)	31(38.75)	5(6.25)
10. Somebody who encourages me to have physical activity regularly.	17(21.25)	32(40)	11(13.75)	17(21.25)	3(3.75)

11. Somebody who reminds me about various methods of physical activity (exercise, job or household activities).	34(42.5)	20(25)	9(11.25)	13(16.25)	4(5)
12. Somebody who pays the cost of registering in a gym or buying equipment for physical activity.	8(10)	14(17.5)	20(25)	29(36.25)	9(11.25)
13. Somebody who reminds me that I must have more physical activity when I am lazy.	33(41.25)	22(27.5)	7(8.75)	14(17.5)	4(5)
14. Somebody who asks me to join him/her in exercising.	4(5)	34(42.5)	16(20)	19(23.75)	7(8.75)
15. Somebody who always asks me about the result of my blood glucose test.	11(13.75)	35(43.75)	10(12.5)	19(23.75)	5(6.25)
16. Somebody who pays attention and reads the amount of my blood glucose from the glucometer during the self-monitoring of blood glucose.	4(5)	15(18.75)	25(31.25)	31(38.75)	5(6.25)
17. Somebody who helps me to monitor my blood glucose with a glucometer when I am not strong enough.	11(13.75)	36(45)	14(17.5)	16(20)	3(3.75)
18. Somebody who reminds me about the time of blood glucose test in a laboratory every 3 months.	7(8.74)	42(52.5)	9(11.25)	16(20)	6(7.5)
19. Somebody who checks all the necessary equipment to perform the Self-Monitoring of Blood Glucose.	5(6.25)	14(17.5)	13(16.25)	36(45)	12(15)
20. Somebody who encourages me to perform the Self-Monitoring of Blood Glucose independently.	7(8.75)	36(45)	11(13.75)	20(25)	6(7.5)
21. Somebody who pays attention to the signs of hypoglycemia in me.	21(26.25)	33(41.25)	15(18.75)	9(11.25)	2(2.5)
22. Somebody who gives me educational materials (CDs, books, etc.) about foot care in diabetics.	13(16.35)	43(53.75)	16(20)	6(7.5)	2(2.5)
23. Somebody who reminds me of the daily foot care.	21(26.25)	37(46.35)	14(17.5)	7(8.75)	1(.25)
24. Somebody who encourages me to perform daily foot care.	30(37.5)	29(36.25)	12(15)	8(10)	1(1.25)
25. Somebody who performs daily foot care for me when I am not strong enough.	33(41.25)	29(36.25)	9(11.25)	7(8.75)	2(2.5)
26. Somebody who always makes sure that all necessary things for foot care such as warm water and mild soap are available.	32(40)	28(35)	10(12.5)	7(8.75)	3(3.75)
27. Somebody who helps me with foot care.	2(2.5)	2(2.5)	2(2.5)	22(27.5)	11(13.75)
28. Somebody who helps and encourages me to quit smoking.	3(3.75)	5(6.25)	8(10)	18(22.5)	5(6.25)
29. Somebody who registers me in a smoke quitting center.	3(3.75)	3(3.75)	4(5)	17(21.25)	12(15)
30. Somebody who gives me educational materials (CDs, books, etc.) about smoking and its effects in diabetics.	3(3.75)	24(30)	17(21.25)	32(40)	2(2.5)

n = number of participants; % = percentage of total responses.
